# A Refined Model for the TSG-6 Link Module in Complex with Hyaluronan: USE OF DEFINED OLIGOSACCHARIDES TO PROBE STRUCTURE AND FUNCTION*

**DOI:** 10.1074/jbc.M113.542357

**Published:** 2014-01-08

**Authors:** Victoria A. Higman, David C. Briggs, David J. Mahoney, Charles D. Blundell, Benedict M. Sattelle, Douglas P. Dyer, Dixy E. Green, Paul L. DeAngelis, Andrew Almond, Caroline M. Milner, Anthony J. Day

**Affiliations:** From the ‡ Department of Biochemistry, University of Oxford, Oxford OX1 3QU, United Kingdom,; § Wellcome Trust Centre for Cell Matrix Research,; ¶ Faculty of Life Sciences, University of Manchester, Oxford Road, Manchester M13 9PT United Kingdom, and; the ‖ Department of Biochemistry and Molecular Biology, Oklahoma Center for Medical Glycobiology, University of Oklahoma Health Sciences Center, Oklahoma City, Oklahoma 73104

**Keywords:** Chondroitin, Hyaluronate, Isothermal Titration Calorimetry, Molecular Modeling, NMR, Defined Sugars, TSG-6

## Abstract

Tumor necrosis factor-stimulated gene-6 (TSG-6) is an inflammation-associated hyaluronan (HA)-binding protein that contributes to remodeling of HA-rich extracellular matrices during inflammatory processes and ovulation. The HA-binding domain of TSG-6 consists solely of a Link module, making it a prototypical member of the superfamily of proteins that interacts with this high molecular weight polysaccharide composed of repeating disaccharides of d-glucuronic acid and *N*-acetyl-d-glucosamine (GlcNAc). Previously we modeled a complex of the TSG-6 Link module in association with an HA octasaccharide based on the structure of the domain in its HA-bound conformation. Here we have generated a refined model for a HA/Link module complex using novel restraints identified from NMR spectroscopy of the protein in the presence of 10 distinct HA oligosaccharides (from 4- to 8-mers); the model was then tested using unique sugar reagents, *i.e.* chondroitin/HA hybrid oligomers and an octasaccharide in which a single sugar ring was ^13^C-labeled. The HA chain was found to make more extensive contacts with the TSG-6 surface than thought previously, such that a d-glucuronic acid ring makes stacking and ionic interactions with a histidine and lysine, respectively. Importantly, this causes the HA to bend around two faces of the Link module (resembling the way that HA binds to CD44), potentially providing a mechanism for how TSG-6 can reorganize HA during inflammation. However, the HA-binding site defined here may not play a role in TSG-6-mediated transfer of heavy chains from inter-α-inhibitor onto HA, a process known to be essential for ovulation.

## Teaser

**Background:** The polysaccharide hyaluronan is organized through interactions with the protein TSG-6 during inflammation and ovulation.

**Results:** NMR spectroscopy on TSG-6 in the presence of defined sugars provided restraints that allowed modeling of a refined hyaluronan/TSG-6 complex.

**Conclusion:** TSG-6 binding causes bending of hyaluronan that explains its condensation of this polysaccharide.

**Significance:** This provides novel structural insights into protein-hyaluronan interactions.

## Introduction

Hyaluronan (HA)^5^ is a linear high molecular weight glycosaminoglycan consisting of repeating disaccharides of β4-d-glucuronic acid (GlcUA) and β3-*N*-acetyl-d-glucosamine (GlcNAc), which is ubiquitously present in the extracellular matrix (ECM) of vertebrate tissues. It plays many different and important biological roles such as providing structural organization to the ECM and regulating cell mobility/activity in the context of both physiological process and disease (1–4). This diversity of function has been suggested to arise via the interaction of HA with various HA-binding proteins (termed hyaladherins) leading to the formation of multimolecular complexes with distinct structural arrangements (or architectures) that likely underlie their different functions (5–7). Most hyaladherins belong to a superfamily of proteins that interact with HA via HA-binding domains (HABD) composed of Link modules, where these have been subdivided into three groups (A, B, and C) on the basis of the size of the HABDs (7, 8). The smallest HABD, denoted as “type A,” consists of an individual Link module domain, for which both NMR and crystal structures have been determined for this ∼100-amino acid region of human tumor necrosis factor-stimulated gene-6 (TSG-6) (9–11); the Link module was revealed to be a compact structure composed of two β-sheets (I and II) flanked by two α-helices with a fold related to that of the C-type lectin domain. Currently, the ∼150-residue HABD from mouse and human CD44, a major cell surface receptor for HA, provides the only other high resolution structural information for a member of the Link module superfamily (12, 13). In this “type B” HABD, N- and C-terminal sequences flanking the Link module extend the β-sheet structure (adding an additional 4 β-strands to the triple-stranded βI-sheet) to form an extra lobe of structure in intimate contact with the Link module (12); LYVE-1, a related cell surface protein that may function as a HA-binding protein on lymph vessel endothelium and macrophages, is also likely to have a similar extended structure (14). Type C HABDs are composed of contiguous pairs of Link modules (7, 8); however, at present no NMR or crystal structures have been determined. This type of HABD is found in the lectican (aggrecan, brevican, neurocan, and versican) and link proteins (HAPLN1–4), where both modules are necessary for folding and are believed to contribute to HA binding; in some cases an N-terminal immunoglobulin-like fold may also contribute structural stability (15, 16). Although no high resolution data have been obtained for the type C HABDs, homology modeling based on the solution structure of the TSG-6 Link module (in its HA-bound conformation) has provided some useful insights into how the contiguous Link modules may pack together (17).

TSG-6 is a 35-kDa HA-binding protein that is usually expressed in response to proinflammatory stimuli at sites undergoing ECM remodeling (18, 19). It has been hypothesized to be an important protector of tissue structure during inflammation. In this regard, TSG-6 has been found to be chondroprotective in murine models of inflammatory arthritis (see Ref. 20), where its serum concentration correlates well with disease severity (21), and has been found to play a potent inhibitor of osteoclast-mediated bone erosion *in vitro* (22, 23); it is also cardioprotective during myocardial infarction (24), can reduce inflammatory damage to the cornea following injury (25), and can attenuate zymosan-induced peritonitis by decreasing proinflammatory signaling in resident macrophages (26). Given that TSG-6 interacts with a large number of ligands (18, 19), not all of these tissue-protective activities are likely to be mediated via its HA-binding function. Nevertheless, TSG-6 has been shown to enhance/induce the interaction of HA with CD44 on lymphocyte cell lines, which could serve to regulate leukocyte migration by promoting cell adhesion/rolling (27, 28). The recent finding that TSG-6 can directly cross-link HA chains, *i.e.* via the formation of HA-induced TSG-6 oligomers (28, 29), provides a mechanism for this whereby TSG-6/HA complexes could, for example, promote CD44 clustering (27) and/or switch this receptor to its high affinity conformation (13). TSG-6-mediated cross-linking of HA could also serve to remodel ECM, which may contribute to its protective anti-inflammatory activities (27).

In addition to its inflammation-associated functions, it is well established that TSG-6 is essential for female fertility in mice, being required for the assembly of a HA-rich ECM around the oocyte prior to ovulation (30, 31). This process, termed cumulus matrix expansion, has been shown to be reliant on the formation of complexes of HA with heavy chains (HC) from the serum proteoglycan inter-α-inhibitor (IαI) (32–34), where TSG-6 acts as a cofactor and catalyst in the covalent transfer of the HCs onto HA (35); TSG-6-mediated formation of HC·HA also occurs at sites of inflammation (36–38). Covalent HC·TSG-6 complexes act as intermediates in HC transfer (35, 39, 40); this transesterification reaction is preceded by the formation of non-covalent complexes between TSG-6 and IαI that inhibit the HA-binding activity of TSG-6, prevent/disrupt HA-cross-linking, and promote TSG-6 catalytic function (28). The HCs are then transferred from TSG-6 onto HA (35), presumably when the HC·TSG-6 complex and HA interact, although the details of this are not yet fully understood. As well as catalyzing HC·HA production, TSG-6 perhaps may contribute to the stabilization of the cumulus ECM through its simultaneous interaction with HA and pentraxin-3 (41, 42), *i.e.* a multimeric protein (43) that has been implicated as being essential for female fertility (41, 44); IαI HCs also interact with PTX3, providing a possible mechanism for the cross-linking of HC·HA (45).

The HA-binding site in the recombinant Link module of human TSG-6 (termed Link_TSG6) has been defined by site-directed mutagenesis (27, 46, 47) such that functionally important residues were found to line a shallow binding groove when mapped onto Link_TSG6 in its HA-bound solution conformation (10). Two tyrosine residues were concluded to form aromatic stacking interactions with sequential rings in the sugar. This observation along with the polarity of the HA in the binding groove (determined from a comparison of NMR spectra of Link_TSG6 in the presence of HA oligomers of different length; Ref. 10) formed the basis for modeling of Link_TSG6 in complex with an HA octasaccharide (HA_8_) (17). In this model only 5 of the 8 sugar rings contacted the protein surface (*e.g.* stabilized by salt bridges in addition to π-stacking interactions), whereas a 7-mer (with GlcUA at either end) was the minimal length of HA oligosaccharide to bind with maximum affinity (10). Furthermore, the oligomer was found to adopt a rather linear conformation in the Link_TSG6/HA_8_ model, whereas the more recently determined crystal structure of the murine CD44 HABD in complex with HA_8_ showed the HA oligosaccharide to bend around the protein surface (13). Interestingly, NMR spectra of ^13^C,^15^N-labeled HA_8_ bound to Link_TSG6 contained more than the expected number of peaks, consistent with the octasaccharide being present in more than one conformation (17).

Here we have generated a refined model for the Link module from human TSG-6 in complex with HA by identifying novel restraints based on NMR spectroscopy of Link_TSG6 in the presence of HA oligosaccharides of different length. The model, which was validated by a combination of interaction analysis and NMR experiments with unique sugar reagents (*i.e.* chondroitin/HA hybrid oligomers and an octasaccharide with only one ring isotopically labeled) reveals the HA to make more extensive contacts with the protein surface than thought previously. In particular, the GlcUA at ring 1 of the 8-mer makes a stacking interaction with His^45^ and a salt bridge to Lys^68^ of Link_TSG6, thereby causing the HA to bend around two faces of the Link module; *i.e.* resembling the way HA fits into the binding groove of CD44. This protein-induced conformational perturbation of HA may help explain, at least in part, how TSG-6 can reorganize HA-rich matrices, *e.g.* during inflammation. Furthermore, comparison of the affinities of the interactions of the various HA oligosaccharides with Link_TSG6 with their substrate activities in TSG-6-mediated HC transfer onto HA indicates that the HA-binding surface in the HC·TSG-6 complex is likely to be distinct from that in free TSG-6. Thus the refinement of the Link_TSG6/HA_8_ complex described here provides new and important insights into the structure and function of this archetypal HA-binding domain.

## EXPERIMENTAL PROCEDURES

### 

#### 

##### Preparation of Protein and Sugar Reagents

Unlabeled and uniformly ^15^N-labeled Link_TSG6 (residues 36–133 in the TSG-6 preprotein; Ref. 48) were expressed in *Escherichia coli* and purified as described previously (49–51). HA oligosaccharides of defined length (*i.e.* HA_8_^AN^ (where A and N correspond to GlcUA and GlcNAc at the non-reducing and reducing termini, respectively), HA_7_^AA^, HA_7_^NN^, HA_6_^AN^, HA_6_^NA^, HA_5_^AA^, HA_5_^NN^, HA_4_^AN^, and HA_4_^NA^) were prepared as described in Blundell and Almond (52), and HA/chondroitin chimeric oligosaccharides (HA_4_C_4_ and C_4_HA_4_; with HA_4_^AN^ at reducing and non-reducing termini, respectively) were made as before (53, 54). Chondroitin octasaccharides (C_8_^AN^) were made by limited digestion of chondroitin from *Pasteurella multocida* (55) by bovine testicular hyaluronidase (Calbiochem). Size defined oligosaccharides were purified using DEAE HPLC chromatography with an ammonium bicarbonate gradient, as described in Mahoney *et al.* (56). The C_8_^AN^ elution position was determined by comparison with HA oligosaccharide standards.

HA_8_^AN^-^13^C_6_-GlcUA3 (*i.e.* an HA_8_^AN^ oligomer in which the ring 3 GlcUA is uniformly ^13^C-labeled) was prepared as in DeAngelis *et al.* (54) using UDP-^13^C_6_-GlcUA at the single step needed to insert this NMR-active sugar; uniformly labeled UDP-^13^C_6_-GlcUA was synthesized by oxidizing UDP-^13^C_6_-Glc (Biosupplies Australia Pty Ltd, Victoria, Australia) with the recombinant *E. coli*-derived histidine_6_-tagged version of the streptococcal UDP-Glc dehydrogenase (57) *in vitro*. The immobilized enzyme reactor method was employed as before, but the intermediate oligosaccharides were purified by gel filtration on a P2 column (Bio-Rad) before and after the addition of the labeled sugar to ensure placement of the ^13^C_6_-GlcUA3 only at the desired position.

NMR samples were prepared from lyophilized material reconstituted in 10% (v/v) D_2_O and 0.02% (w/v) NaN_3_; the pH was adjusted to pH 6.0 using NaOH and HCl solutions. In most cases oligosaccharides were added to the ^15^N-labeled Link_TSG6 protein in a 2-fold molar excess so as to ensure complete binding; HA_4_ oligosaccharides were added to ^15^N-Link_TSG6 in a 10-fold molar excess to reflect the lower binding affinities (10), and HA_8_^AN^-^13^C_6_-GlcUA3 was combined with Link_TSG6 at an ∼1:3.3 protein:sugar ratio (based on relative peak heights for free and bound sugar (for the C2, C3, C4, and C5 carbons) in the ^1^H, ^13^C HSQC described below).

##### Nuclear Magnetic Resonance

NMR experiments were performed either on a home-built spectrometer at the Oxford Centre for Molecular Sciences with a ^1^H operating frequency of 599 MHz or on an 800-MHz Bruker instrument at the Department of Molecular Biology and Biotechnology, University of Sheffield. ^1^H,^15^N HSQC spectra were recorded at 25 °C (599 MHz) on ^15^N-labeled Link_TSG6 in the presence of the following HA oligosaccharides: HA_8_^AN^ (0.3 mm protein concentration, 1:2 molar ratio of protein to oligosaccharide), HA_7_^AA^ (0.3 mm, 1:2), HA_7_^NN^ (0.3 mm, 1:2), HA_6_^AN^ (1.0 mm, 1:2), HA_6_^NA^ (2.0 mm, 1:2), HA_5_^AA^ (0.2 mm, 1:1), HA_5_^NN^ (0.3 mm, 1:2), HA_4_^AN^ (0.3 mm, 1:10), HA_4_^NA^ (0.3 mm, 1:10), HA_4_C_4_ (0.25 mm, 1:1.1), and C_4_HA_4_ (0.15 mm, 1:1.1). ^13^C,^1^H HSQC spectra were recorded at 25 °C (800 MHz) on HA_8_^AN^-^13^C_6_-GlcUA3 in the absence (2.5 mm) and presence of unlabeled Link_TSG6 (0.75 mm, 1:3.3). All spectra were processed using NMRPipe (58) and analyzed using Sparky (T. D. Goddard and D. G. Kneller, University of California, San Francisco). The C1-C5 carbons in the free sugar were uniquely assigned using residual ^2^*J*_CH_ cross-peaks, where the chemical shift values for the carbons and their associated protons were found to be consistent with those of internal GlcUA residues in the context of a uniformly ^13^C-labeled HA octasaccharide (*i.e.* with all carbons on all eight sugar residues labeled) analyzed previously (59); although the new peaks, seen in the presence of Link_TSG6, were not independently assigned, the comparison of the ^13^C and ^1^H chemical shifts of the new resonances with those of the free sugar allowed their confident identification.

##### Isothermal Titration Calorimetry

The interactions between Link_TSG6 and HA_8_^NA^, HA_7_^AA^, HA_7_^NN^, HA_6_^NA^, HA_5_^NN^, HA_4_C_4_, C_4_HA_4_, and C_8_^AN^ oligomers were investigated on a Microcal VP-ITC instrument at 25 °C in 5 mm MES, pH 6.0, as described previously (46, 47). Oligosaccharide solutions (0.21–0.32 mm) were added to the protein (0.015–0.029 mm) in 26 × 5-μl injections. The Origin software package was used to fit the data to a one-site model by nonlinear least squares regression after subtracting the heats resulting from the addition of oligosaccharide into buffer alone. Affinities/thermodynamics for the interactions were determined by averaging the results from three separate experiments. ITC data (including previously unpublished thermodynamic values) for HA_10_^AN^, HA_8_^AN^, HA_6_^AN^, and HA_5_^AA^ were taken from Blundell *et al.* (10), and minor numerical errors were corrected; in the case of HA_7_^AA^, only values from the present study were used.

##### Model Building

Models of the Link_TSG6/HA_8_^AN^ complex were built as described previously (17) except for the addition of two extra structural restraints; the coordinates of the 20 lowest energy structures determined for the bound conformation of Link_TSG6 were used (PDB 1o7c; Ref. 10) and were kept fixed throughout, whereas all the saccharide rings were modeled in the ^4^C_1_ conformation. On the basis of the observed HA oligomer-induced NMR shift changes (determined here) as well as recent pH dependence and mutagenesis data (60), an additional 3.5 Å ring-stacking interaction between the ring 1 GlcUA and His^45^ was introduced. This restraint caused distortions in some models that were removed in all but two of the 20 lowest energy structures upon the addition of a further restraint between the ring 4 GlcNAc methyl group and the Ile^61^ side chain (*i.e.* present within a hydrophobic pocket on the Link module surface; Ref 10).

##### Heavy Chain Transfer Assays

The ability of HA oligosaccharides (HA_14_ (control), HA_8_^AN^, HA_8_^NA^, HA_7_^NN^, HA_7_^AA^, HA_6_^AN^, HA_6_^NA^, HA_5_^AA^, HA_5_^NN^, HA_4_^AN^, and HA_4_^NA^) to act as substrates for the covalent attachment of HCs (from IαI) was determined using the assay described before (35); recombinant human TSG-6 was expressed in *Drosophila* Schneider-2 cells and purified as described previously (61), and IαI (a kind gift from Professor Erik Fries) was purified from human serum (62). Briefly, IαI (320 μg/ml), TSG-6 (80 μg/ml), and HA oligosaccharides (molar equivalents of the HA_14_ control at 40 μg/ml) were incubated in 20 mm HEPES-HCl, pH 7.5, 150 mm NaCl, 5 mm MgCl_2_ in a total volume of 25 μl for 2 h at 4 °C. Samples (15 μl) were then run on 10% (w/v) Tris-Tricine/SDS-polyacylamide gels after reduction with 5% (v/v) β-mercaptoethanol in SDS protein sample buffer (5 min at 100 °C) and stained using Coomassie Blue. All HA oligosaccharides were analyzed at least three times, and the relative intensities of the various HC·HA*_n_* species formed (∼85 kDa) were determined by eye.

## RESULTS

### 

#### 

##### Defining the Position of HA within the Binding Groove of the TSG-6 Link Module

In a previous study we generated a model of the TSG-6 Link module (Link_TSG6) in complex with an HA_8_^AN^ oligosaccharide (17). This model was based in part on the solution structure of Link_TSG6 in the presence of HA_8_^AN^ (*i.e.* in its HA-bound conformation) and on the comparison of ^1^H,^15^N HSQC spectra of ^15^N-labeled Link_TSG6 in the presence of unlabeled HA oligosaccharides of different length (10); *i.e.* HA_10_^AN^, HA_8_^AN^, HA_7_^AA^, HA_6_^AN^, HA_5_^AA^, or HA_4_^AN^. The spectra were very similar and showed the different oligosaccharides to be lying in the same binding site on Link_TSG6. A few discrete differences in the spectra were used to deduce the orientation and approximate positioning (register) of the octasaccharide on the Link_TSG6 HA-binding surface. This and other information was used to create a model of the Link_TSG6/HA_8_^AN^ complex (17).

**FIGURE 1.**
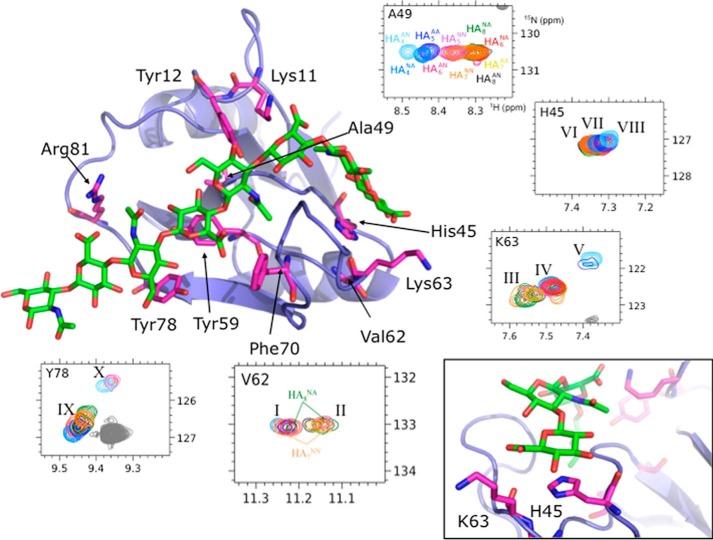
**Use of NMR spectroscopy to refine the model of the Link_TSG6/HA_8_^AN^ complex.** ^1^H,^15^N HSQC NMR spectra were acquired for ^15^N-labeled Link_TSG6 in the presence of 10 different HA*_n_* oligosaccharides. Regions of these spectra are shown for backbone amide resonances (color-coded as defined for the Ala^49^ shift map) that experience differential perturbations compared with HA_8_^AN^; distinct sets of resonances are sub-grouped accordingly using Roman numerals. The positions of these differentially perturbed residues (side chain carbons in *pink*) are shown on the Link_TSG6 NMR structure (*blue ribbon*) in its HA_8_^AN^-bound conformation (PDB accession code 1o7c; Ref. 10); the lowest energy structure from the family of 20 structures is used here and in all other figures. HA_8_^AN^ (carbons are in *green*) is displayed in a representative Link_TSG6-bound conformation, *i.e.* as determined in this study from the refined model of the Link_TSG6/HA_8_^AN^ complex; this particular HA_8_^AN^ conformer is used in all subsequent figures. The *inset* (*bottom right corner*) shows the ring stacking interaction between the non-reducing terminal glucuronic acid (ring 1) and His^45^, inferred from the refined Link_TSG6/HA_8_^AN^ model, and the proximity of this sugar ring to Lys^63^, with which it may form a salt bridge; in this particular model the Lys^63^ is not in the correct orientation to make such an ionic interaction.

Since then a new set of HA oligosaccharides that contain a GlcNAc at their non-reducing termini have become available (52). Further ^1^H,^15^N HSQC spectra of ^15^N-labeled Link_TSG6 with these new HA oligosaccharides were recorded so as to test the previous model. These were found to be very similar to the previous NMR spectra, indicating that the oligosaccharides are all binding to Link_TSG6 at the same site. However, for several residues key differences in the chemical shift values of their backbone amide resonances were seen. Fig. 1 shows overlays of the ^1^H,^15^N HSQC spectra for some of these residues in Link_TSG6 when it is in complex with the 10 different HA oligosaccharides possible for HA_4_ to HA_8_ (*i.e.* including the 5 new oligomers described under “Experimental Procedures”).

**FIGURE 2.**
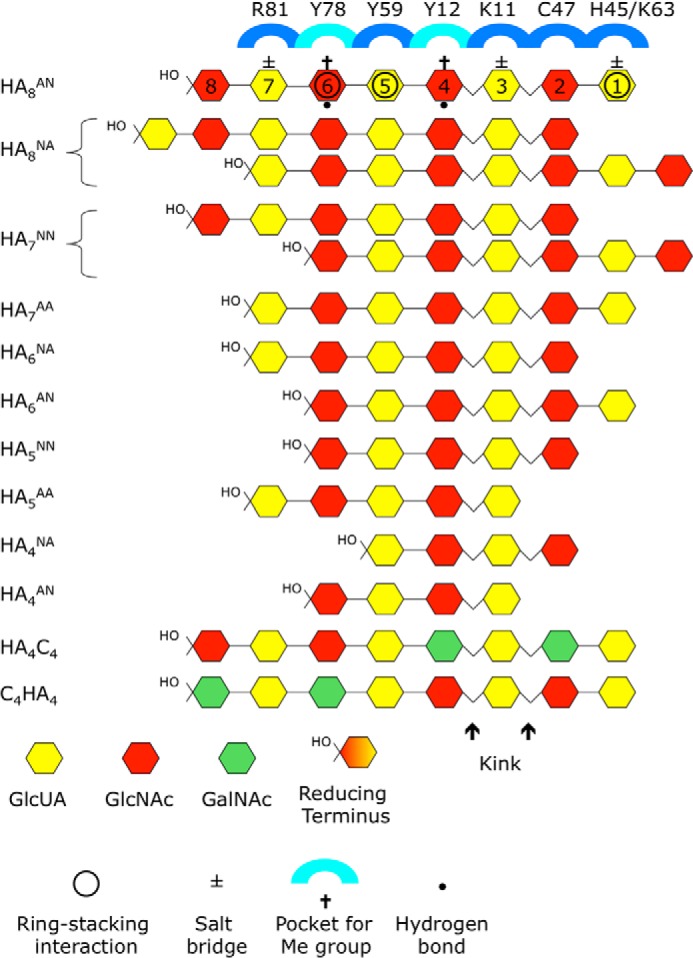
**Schematic showing how oligosaccharides are accommodated in HA-binding groove of Link_TSG6.** Ten HA oligomers ranging in length from tetra- to octasaccharides and two-hybrid (chondroitin/HA) 8-mers were used in the current study. Differences in NMR chemical shift perturbations of Link_TSG6 in the presence of these oligomers were used to determine their orientation and register within the HA-binding site as well as their positions relative to key amino acids that mediate the interaction. This analysis indicates that the HA_8_^AN^ oligosaccharide interacts with Link_TSG6 through a combination of ionic interactions (*i.e.* between Lys^11^, Lys^63^, and Arg^81^ and rings 3, 1, and 7 of the sugar, respectively), ring-stacking interactions (between His^45^, Tyr^59^, and Tyr^78^ and rings 1, 5, and 6), hydrogen bonds (between the hydroxyls of Tyr^12^ and Tyr^78^ and rings 4 and 6), and accommodation of the methyl groups from rings 4 and 6 (GlcNAc sugars) in hydrophobic pockets at the bottom of the binding groove; rings 2 and 3 wrap round the Cys^47^-Cys^68^ disulfide, making van der Waals contacts (where Cys^47^ is in close proximity to ring 2), and the Phe^70^ may also make an occasional ring stacking interaction with ring 4 (not shown). From modeling of the HA_8_^AN^ into the structure of Link_TSG6 (determined in its HA_8_^AN^-bound conformation), it is apparent that the oligosaccharide must kink (*i.e.* at the glycosidic bond between rings 2 and 3) to become fully accommodated within the binding site.

Two distinct sets of peaks are seen within the spectra of Val^62^. One arises from Link_TSG6 in complex with HA_8_^AN^, HA_7_^AA^, and HA_6_^AN^ (denoted by *II* on Fig. 1), and the other arises from HA_6_^NA^ and the four HA_5_ and HA_4_ oligosaccharides (*peak I*); HA_8_^NA^ and HA_7_^NN^ have peaks in both positions. Thus a particular chemical shift perturbation is seen at Val^62^ for all oligosaccharides that have a GlcUA at position 1 (Fig. 2). The spectra also provide evidence for HA_8_^NA^ and HA_7_^NN^ binding to Link_TSG6 in two different registers, *e.g.* with the reducing terminal ring of HA_7_^NN^ placed at either position 8 (analogous to HA_8_^AN^) or position 6 (analogous to HA_6_^AN^); two registers were seen previously for HA_10_^AN^ (10).

The spectra for Lys^63^ contains three main peak clusters: peak III (on Fig. 1) arising from HA_8_^NA^, HA_8_^AN^, HA_7_^NN^, HA_7_^AA^, and HA_6_^AN^, peak IV from HA_8_^NA^, HA_7_^NN^, HA_6_^NA^, HA_5_^NN^, and HA_4_^NA^, and peak V from HA_5_^AA^ and HA_4_^AN^. These data show that the environment of Lys^63^ is sensitive to the presence or absence of a GlcUA ring at position 1 as well as to the presence or absence of a GlcNAc ring at position 2 (see Fig. 2). The double register of HA_8_^NA^ and HA_7_^NN^ is again apparent (being present in peaks III and IV; Fig. 1), and the influence of the extra GlcNAc ring at the non-reducing terminus in one of these conformations is also visible in the Lys^63^ shift map by the slight downfield shift of the peak relative to the other resonances in both the ^1^H and ^15^N dimensions. Furthermore, small exchange peaks show that the sugar moves between one register and the other on the millisecond timescale of the NMR experiment. His^45^ gives rise to a similar pattern of resonances to Lys^63^, *i.e.* with three sets of peaks (VI, VII, and VIII) but with shifts of smaller magnitude; the NH resonance for Cys^47^ is also perturbed in a similar manner (data not shown).

The orientation of the HA oligosaccharides can be inferred from the shift perturbations for Ala^49^ and Tyr^78^ (Fig. 1). Both draw a distinction between oligosaccharides containing a GlcUA ring at position 7 (HA_8_^AN^, HA_8_^NA^, HA_7_^NN^, HA_7_^AA^, HA_6_^NA^, HA_5_^AA^, *e.g.* corresponding to peak IX on Fig. 1) and those terminating with a GlcNAc at position 6 (HA_7_^NN^, HA_6_^AN^, HA_5_^NN^, HA_4_^AN^ corresponding to peak X). In addition, the Ala^49^ shift map shows distinct peaks for HA_5_^AA^, HA_4_^NA^, and HA_4_^AN^. These peaks may arise from the lower binding affinity of these oligosaccharides (see ITC results below) that allows greater flexibility to the β4-β5 loop in Link_TSG6 (10) and a consequent averaging of chemical shifts.

##### Identifying New Interactions between HA and the TSG-6 Link Module

The ^1^H,^15^N HSQC spectra of Link_TSG6 with the new HA oligomers are all consistent with the previous binding model (17), including the orientation and register of the oligosaccharides within the Link module binding groove (summarized in Fig. 2). However, this comparative shift map analysis has identified novel perturbations to His^45^, Val^62^, and Lys^63^ when ring positions 1 or 2 are occupied. Thus, these residues are likely to be involved in protein-oligosaccharide interactions or at least be in close proximity to the bound HA. This might explain how HA_7_^NN^ is able to bind in two registers and why HA_6_^AN^ binds at positions 1–6 rather than 3–8. In both cases the salt bridge at Arg^81^ is omitted in favor of binding at positions 1 and 2, suggesting that the sugar is in fact able to form favorable interactions with the protein at these points. In this regard, inspection of the Link_TSG6 structures in the presence and absence of HA_8_^AN^ (10, 11) shows that His^45^ lies flat on the surface of the protein when it is bound to HA_8_^AN^, in contrast to the free protein, where it is at right angles to the surface and partially buried. Thus a ring stacking interaction between this residue and a sugar ring would be possible, where this is likely to be affected by the charge state of the histidine ring; histidine side chains have been shown to be able to make π-stacking interactions with both protein and non-protein ligands (63, 64). It has been shown that the Link_TSG6/HA interaction is pH-dependent and that one of the contributing factors to this is the protonation/deprotonation of His^45^, the deprotonated form being required for maximal binding (60); the p*K_a_* of His^45^ is 5.7 in the HA_8_^AN^-bound protein, and this residue was demonstrated to be uncharged at pH 6.0 (*i.e.* under the conditions used here). Furthermore, mutation of His^45^ to serine causes a greater than 2-fold reduction in the binding affinity (60).

##### New Model for the Link_TSG6/HA_8_^AN^ Complex

**FIGURE 3.**
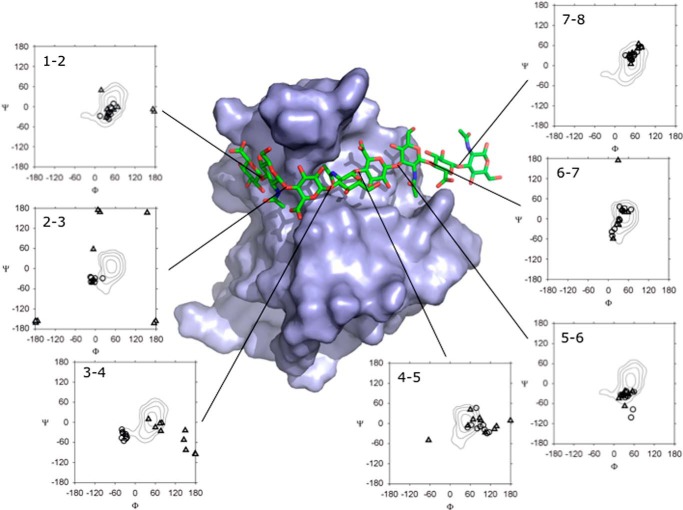
**Glycosidic bond angles for family of 20 models determined for Link_TSG6/HA_8_^AN^ complex.** The Link_TSG6 protein structure is shown as a solvent-accessible surface with the modeled HA_8_^AN^ oligosaccharide in a representative conformation (see legend to Fig. 1). Φ/Ψ space plots are displayed for the 7 glycosidic linkages (*i.e.* between rings 1 and 2 (1–2), 2 and 3 (2–3), etc.) in the final 20 models. Glycosidic bond angles are denoted by either *circles* for the 10 best models, *i.e.* that lie within the most favored regions of the Φ/Ψ space plot or by *triangles* for the 10 models that were rejected (*i.e.* based on the presence of distorted bonds or glycosidic angles outside these favorable regions); the contour plots describe the preferred conformations predicted by molecular dynamic simulations for HA in solution (61–63). The contours levels shown are at 2 kcal/mol intervals above the lowest energy.

**FIGURE 4.**
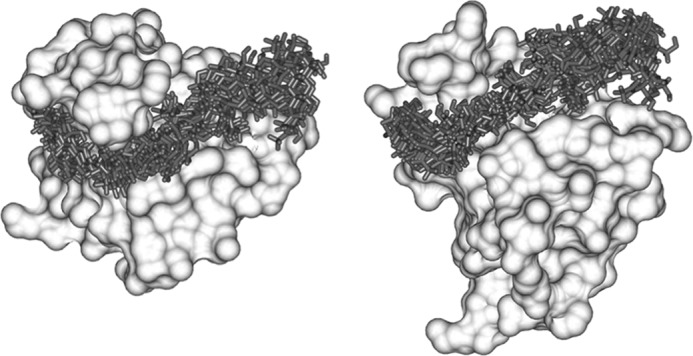
**Family of models of the Link_TSG6/HA_8_^AN^ complex.** Shown is an overlay of 10 selected models where the *left* and *right hand* images are rotated through ∼90° (around the *x* axis) relative to one another. The HA conformers (*sticks*) are displayed on the lowest energy protein structure (shown as a solvent-accessible surface).

We have now refined the model of the Link_TSG6/HA_8_^AN^ complex to include a ring-stacking interaction between His^45^ and ring 1 of the HA octasaccharide (see “Experimental Procedures”). Although in the initial models generated, the HA was always positioned within the expected binding groove on the surface of the protein, in some cases bond distortions were observed, and the sugar passed through the protein behind the Cys^47^-Cys^68^ disulfide bridge. To prevent these implausible conformations, a restraint was introduced between the *N*-acetyl methyl group of ring 4 and the side chain of Ile^61^. This fixed ring 4 of HA in the position observed previously where the Me group is buried in a small hydrophobic pocket present on the surface of the protein in the HA-bound structure (PDB 1o7c; Ref. 10). Two of the final 20 models generated still contained distorted bonds, and a further eight contained HA glycosidic bond angles well outside the most favored regions of the Φ/Ψ space plot determined previously for HA (65–67); therefore, these models (Fig. 3; *triangles*) were rejected. As can be seen from Fig. 4, the remaining 10 models overlay well (denoted by *circles* on Fig. 3) and contain only two HA glycosidic bond angles outside the favored regions. Thus, this family of 10 related structures constitutes the refined model of the Link_TSG6/HA_8_^AN^ complex, where the HA conformer in association with the lowest energy protein solution structure (*i.e.* from the family of models in Ref. 10) is shown in Figs. 1 and 3. The new model is very similar to that in Blundell *et al.* (17) for rings 3–8; however, rings 1–2 now make close contact with the protein surface via interactions with His^45^ and Lys^63^ (see Fig. 1). These new interactions are possible due to a pronounced kink in the HA chain involving the glycosidic bond between rings 2–3 and 3–4. However, it should be noted that the Φ/Ψ angles adopted all have energetically favored conformations (65–67); the Φ angles in the selected 10 models, which all have a restricted range of values (see Fig. 3), indicate that these sugar rings are adopting a non-exoanomeric conformation. It is likely that this conformation is stabilized by the binding of HA to the Link module, as has been seen for other carbohydrate-protein interactions (see Ref. 68). As described in the following sections, we then tested the validity of this Link_TSG6/HA_8_^AN^ model using a combination of ITC and NMR analyses.

##### Interactions of HA Oligomers with Link_TSG6

**FIGURE 5.**
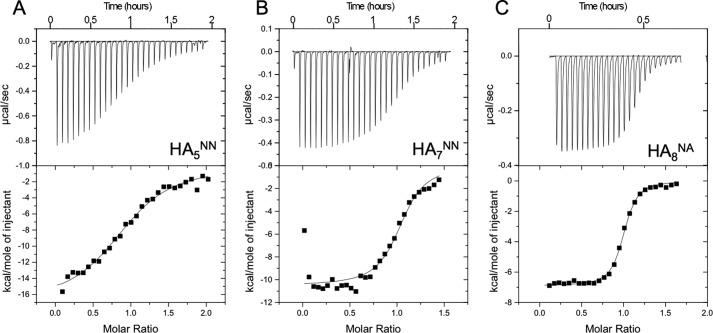
**ITC analysis of the interaction between Link_TSG6 and HA oligomers of different length.** Representative titration plots for the binding of Link_TSG6 to HA_5_^NN^ (*A*), HA_7_^NN^ (*B*), and HA_8_^NA^ (*C*) were determined from the integrated raw data after subtraction of heats of dilution of injectant (*i.e.* oligosaccharide); protein concentrations of 29, 15, and 15 μm and HA concentrations of 318, 210, and 240 μm were used, respectively. For each titration the data are fit by least squares regression to a one-site model, where the derived dissociation constants and stoichiometries are presented in Table 1.

ITC was used to determine the affinities for the interactions between Link_TSG6 and the new HA oligosaccharides (Table 1); representative ITC plots for HA_8_^NA^, HA_7_^NN^, and HA_5_^NN^ are illustrated in Fig. 5. Together with the data determined previously for the other oligomers (see Table 1; Ref. 10), it can be seen that HA_8_^AN^ and HA_10_^AN^ bind with essentially identical affinity and that both 7-mers have only slightly weaker binding (∼70% of HA_8_^AN^); the *K_b_* for the HA_7_^NN^ interaction will be an average of the affinities for the two different binding modes of this oligomer (Fig. 2), which as noted above exchange on the millisecond timescale, making it impossible to dissect the individual affinities corresponding to the two registers. However, the 5- and 6-mers display considerably lower affinity (ranging from ∼5–13% of HA_8_^AN^) revealing that these oligomers all bind sub-optimally to the TSG-6 Link module. From the schematic in Fig. 2, it can be seen that HA_7_^AA^ is the shortest HA oligosaccharide to completely fill the HA-binding site as defined in the refined model of the Link_TSG6/HA_8_^AN^ complex. Unexpectedly, the alternative 8-mer (HA_8_^NA^) binds to Link_TSG6 with an ∼2-fold higher affinity than HA_8_^AN^; as for HA_7_^NN^, this oligomer is present in two different registers, where the 8-mer with a GlcUA at position 1 is the most abundant species (based on NMR peak ratios for Val^62^; see Fig. 1), and therefore, this binding mode will dominate the ITC-derived data. From the Δ*H* and *T*Δ*S* values (Table 1), it is apparent that this increase in affinity is due to a more favorable entropy. Why this oligomer should bind more tightly than HA_8_^AN^ (and HA_10_^AN^) is not clear, and the analyses of the thermodynamic parameters in Table 1 (which have been included here for completeness) are difficult to interpret; deciphering differences in enthalpic and entropic contributions in protein-ligand interactions is far from straightforward (see Ref. 69).

**TABLE 1:** **Binding and thermodynamic constants for the interaction of Link_TSG6 with oligosaccharides of defined size as determined by ITC**

Oligosaccharide	*N*	*K_b_*	Δ*G*	Δ*H*	*T*Δ*S*	% HA_8_^AN^
		× *10^5^m*^−*1*^	*kcal*·*mol*^−*1*^	*kcal*·*mol*^−*1*^	*kcal*·*mol*^−*1*^	
HA_10_^AN^ *^a^*	1.01 ± 0.00	61.2 ± 14.4	−9.19 ± 0.14	−7.57 ± 0.67	1.62 ± 0.60	113
HA_8_^NA^ *^b^*	0.97 ± 0.01	102.3 ± 3.78	−9.56 ± 0.02	−6.90 ± 0.03	2.66 ± 0.03	188
HA_8_^AN^ *^c^*	1.00 ± 0.01	54.3 ± 5.55	−9.16 ± 0.07	−8.01 ± 0.35	1.15 ± 0.38	100
HA_7_^NN^ *^b^*	0.93 ± 0.03	36.8 ± 0.84	−8.96 ± 0.01	−11.26 ± 0.34	−2.30 ± 0.33	67.8
HA_7_^AA^ *^b^*	0.98 ± 0.04	39.2 ± 1.71	−9.00 ± 0.02	−10.03 ± 0.47	−1.03 ± 0.45	72.2
HA_6_^NA^ *^b^*	0.89 ± 0.06	7.0 ± 0.53	−7.96 ± 0.04	−11.29 ± 0.28	−3.33 ± 0.24	12.9
HA_6_^AN^ *^a^*	1.00 ± 0.01	5.8 ± 1.64	−7.77 ± 0.16	−5.40 + 0.11	2.37 ± 0.12	10.6
HA_5_^NN^ *^b^*	1.03 ± 0.02	5.9 ± 0.34	−7.87 ± 0.03	−15.25 ± 0.35	−7.38 ± 0.38	10.8
HA_5_^AA^ *^a^*	1.01 ± 0.02	2.9 ± 1.60	−7.35 ± 0.37	−7.06 ± 1.33	0.30 ± 0.97	5.3
HA_4_^AN^ *^d^*	1	0.22				0.4
C_4_HA_4_ *^b^*	0.91 ± 0.08	33.0 ± 5.44	−8.87 ± 0.11	−7.65 ± 0.10	1.22 ± 0.10	60.8
HA_4_C_4_ *^b^*	0.96 ± 0.06	49.7 ± 4.47	−9.13 ± 0.05	−7.02 ± 0.25	2.11 ± 0.29	91.4
C_8_ *^b^*	0.97 ± 0.07	11.0 ± 1.98	−8.22 ± 0.11	−6.27 ± 0.39	1.95 ± 0.28	20.2

*^a^* Stiochiometry (*N*) and *K_b_* values were taken from Blundell *et al.* (10), and Δ*G*, Δ*H*, and −*T*Δ*S* were calculated from these data; minor corrections to values for HA_10_^AN^, HA_6_^AN^, and HA_5_^AA^ quoted in (10) are based on reanalysis of data.

*^b^* Mean values were determined here from three separate ITC experiments (±S.E.).

*^c^* Mean values determined from eight datasets taken from (10) and two additional ITC experiments (±S.E.).

*^d^* Mean value (taken from Blundell *et al.* (10)) estimated from NMR assuming a 1:1 stoichiometry.

##### Interaction of Chondroitin/HA Hybrid Oligomers with Link_TSG6

**FIGURE 6.**
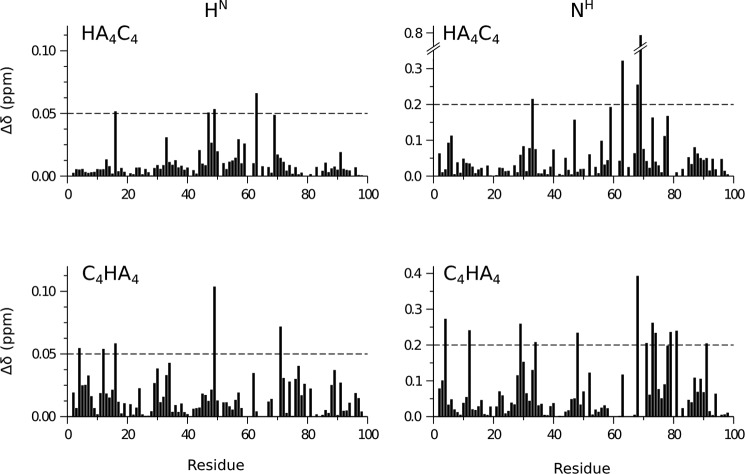
**Chemical shift perturbations in Link_TSG6 caused by hybrid oligomers HA_4_C_4_ and C_4_HA_4_ relative to HA_8_^AN^.** *Bar charts* show the absolute chemical shift differences (Δδ) between Link_TSG6 in the presence of HA_8_^AN^ compared with HA_4_C_4_ (*top*) or C_4_HA_4_ (*bottom*). The Δδ values are shown for the backbone amide protons (H^N^) and nitrogens (N^H^) that were derived from ^1^H,^15^N HSQC spectra acquired on ^15^N-labeled Link_TSG6, where the *horizontal dotted lines* represent the arbitrary threshold levels chosen to indicate a significantly perturbed resonance (H^N^ > 0.05 ppm; N^H^ > 0.20 ppm).

Hybrid GAG polymers can be made using the HA synthase, PmHAS, and the chondroitin synthase, PmCS, from the Gram-negative bacterium *P. multocida* (54). Here two such chimeric oligosaccharides, HA_4_C_4_ and C_4_HA_4_, were used to test the validity of the refined Link_TSG6/HA_8_^AN^ model. Given that the disaccharides of unsulfated chondroitin (-β1,4-GlcUA-β1,3-GalNAc-) and HA (-β1,4-GlcUA-β1,3-GlcNAc-) differ only by the placement of one OH group (at the 4 position), it seemed likely that these oligosaccharides would be accommodated within the Link module HA-binding groove but that any small differences in their binding compared with HA_8_^AN^ might prove informative. In this regard, ^1^H,^15^N HSQC spectra of Link_TSG6 in the presence of HA_4_C_4_ or C_4_HA_4_ were found to be very similar to that of the Link_TSG6/HA_8_^AN^ complex (Fig. 6); thus, the hybrid oligosaccharides both bind to Link_TSG6 at the same site as the HA oligomers, as was anticipated. The differences seen in the chemical shifts induced in Link_TSG6 by HA_4_C_4_ and C_4_HA_4_, in comparison to HA_8_^AN^, are consistent with the orientation and positioning of HA in the refined (and original) models.

**FIGURE 7.**
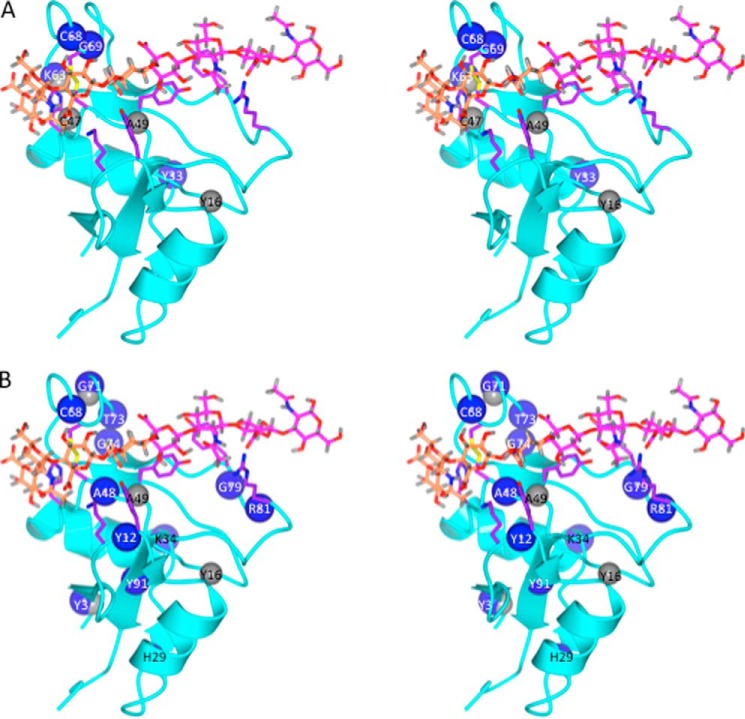
**Mapping of shift perturbations caused by hybrid oligomers HA_4_C_4_ and C_4_HA_4_ onto the refined Link_TSG6/HA_8_^AN^ model.** *A* and *B*, stereoviews of the refined Link_TSG6/HA_8_^AN^ model onto which the amide protons (*gray spheres*) and nitrogens (*blue spheres*) of Link_TSG6 determined to be significantly perturbed by HA_4_C_4_ (*A*) and C_4_HA_4_ (*B*), *i.e.* compared with chemical shift values in the presence of HA_8_^AN^ (see Fig. 6), have been mapped. Amino acids implicated in mediating the interaction with HA are depicted in *purple*, whereas rings 1–4 and 5–8 of HA_8_^AN^ are colored in *orange* and *pink*, respectively. In HA_4_C_4_ (*A*) the chondroitin sugars are positioned at the non-reducing end (*orange*), whereas C_4_HA_4_ (*B*) has its chondroitin sugars positioned at the reducing end (*pink*).

HA_4_C_4_ interacts with Link_TSG6 with an essentially identical affinity to that of HA_8_^AN^ (∼91%); this interaction has an enthalpy term that is 1 kcal·mol^−1^ less favorable than for HA_8_^AN^, which is compensated by a more favorable entropy (see Table 1). In the case of HA_4_C_4_ there are only a few chemical shift perturbations above the threshold that were chosen as significant (*i.e.* 0.05 and 0.2 ppm for H^N^ and N^H^, respectively). The three largest N^H^ shift changes (see Fig. 6) correspond to Lys^63^, Cys^68^, and Gly^69^, and three of the significantly perturbed H^N^ (*i.e.* for Cys^47^, Ala^49^, and Lys^63^) are all in close proximity to the sugar rings 1–4 in the Link_TSG6/HA_8_^AN^ model (*colored orange* on Fig. 7), which includes GalNAc at rings 2 and 4 in this hybrid oligomer. Ring 2 lies above the disulfide bond formed between Cys^47^ and Cys^68^ (that are both perturbed), and Ala^49^ and Gly^69^ are adjacent to ring 4, where these subtle perturbations can be readily explained by the change in position of the C4 hydroxyls from equatorial to axial; for example, Gly^69^ forms part of the specificity pocket occupied by the *N*-acetyl moiety of ring 4 (17), where a change in orientation of this group, *e.g.* caused by a steric clash between Tyr^12^ and the C4-OH in the hybrid oligomer (and the loss of a hydrogen bond between the hydroxyl Hη proton of Tyr^12^ and the C4-OH of HA; Ref. 10), could induce the large chemical shift perturbation observed. The only other significant shift perturbation to an amide nitrogen is in Tyr^33^, which is distant from the HA-binding groove, where we have shown previously that this atom is sensitive to changes in salt strength (70). Therefore, it seems plausible that this perturbation results from the presence of counter ions in this preparation; the shift change observed for the H^N^ of Tyr^16^ (with both hybrid oligosaccharides) may also result from minor changes in buffer composition. Therefore, the chemical shift differences seen in the Link_TSG6 NMR spectra in the presence of HA_4_C_4_ compared with HA_8_^AN^ are consistent with the orientation of HA in the refined and original models.

The C_4_HA_4_ oligomer binds with a somewhat lower affinity to Link_TSG6 compared with that of HA_8_^AN^ (∼61%); *i.e.* due to a decreased enthalpy (see Table 1). The generally small perturbations observed in the ^1^H,^15^N HSQC are more widespread than for HA_4_C_4_ described above (Fig. 7). Although Gly^79^ and Arg^81^ (which have perturbed N^H^) are located near to sugars 5–8 (colored *pink* in Fig. 7), none of the other differentially affected residues are in close proximity to rings 6 and 8. However, the majority of these amino acids are in regions of Link_TSG6 that have been found previously to undergo significant conformational changes on HA binding (*e.g.* β1-α1 loop (Tyr^12^) and β4-β5 loop (Cys^68^, Gly^71^, Thr^73^ and Gly^74^)) (10). It seems likely, therefore, that the conformational change induced by C_4_HA_4_ is slightly different from that caused by HA_8_^AN^, *i.e.* due to a subtle alteration in the position of this hybrid oligomer within the binding groove. This might disrupt the hydrogen bond between the side-chain OH group of Tyr^78^ (that has a slowly exchanging Hη in the presence of HA_8_^AN^; Ref. 10) and the C4 OH group on ring 6, and this could give rise to the lower affinity for C_4_HA_4_. This conformational difference may also explain the perturbation to H^N^ of Ala^49^ that lines the specificity pocket occupied by ring 4. Furthermore, the slight reduction in binding affinity and the lack of major chemical shift perturbations seen with the C_4_HA_4_ oligomer indicate that the stacking interaction between Tyr^78^ and ring 6 can still take place when a GlcNAc is replaced by a GalNAc. This is consistent with the revised (and original) Link_TSG6/HA_8_^AN^ models, in which the face of the GlcNAc of ring 6 that stacks against the side chain of Tyr^78^ is correctly predicted as only one face of GalNAc can make such a ring stacking interaction (due to steric hindrance of the other face by the axial C4-OH group). The other perturbations (to Tyr^3^, His^29^, Lys^34^, and Tyr^91^), although difficult to rationalize, may result from long-range conformational effects or C_4_HA_4_-induced changes in dynamics; *e.g.* we have described previously that Lys^54^, although distant from the ligand binding groove, becomes less dynamic on interaction with HA (11).

The *K_b_* of a chondroitin 8-mer was found to be ∼20% of that for HA_8_^AN^. This indicates that when a GalNAc is present at all GlcNAc positions the combination of the deleterious effects seen for the hybrid oligomers (described above) causes a substantial reduction in affinity, in part due to the loss of favorable enthalpic interactions (see Table 1). This is perhaps because of the loss of hydrogen bonds between C4 hydroxyls on rings 4 and 6 and Tyr^12^ and Tyr^78^, respectively; Ref. 10).

##### Interactions of Labeled-HA with Link_TSG6

**FIGURE 8.**
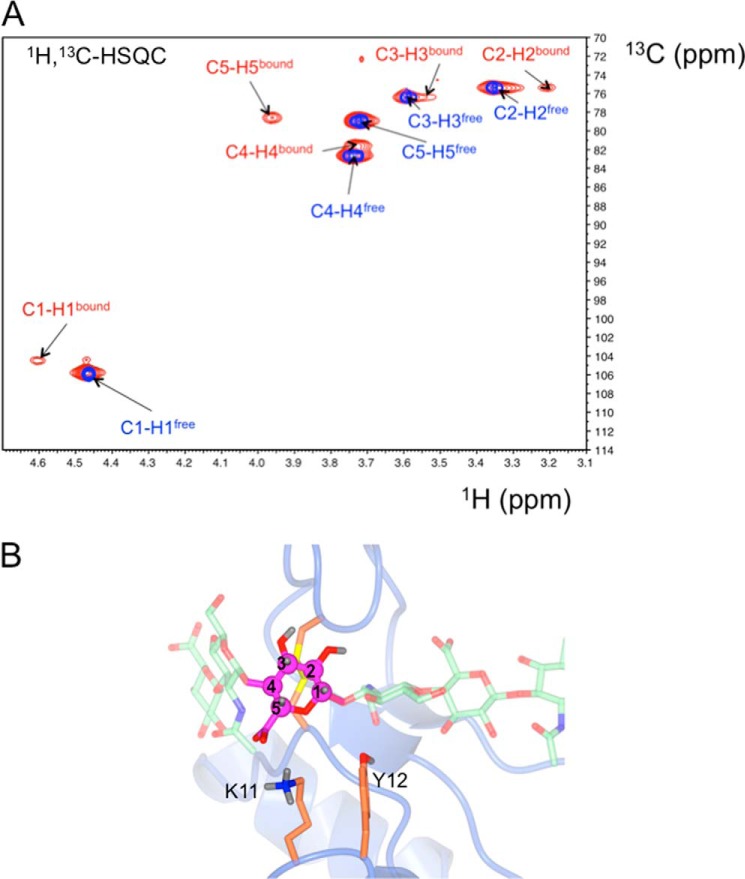
**Testing the Link_TSG6/HA_8_^AN^ model by NMR with a ^13^C-labeled HA 8-mer.** *A*, an overlay of ^13^C,^1^H HSQC NMR spectra of an HA_8_^AN^-oligosaccharide in which ring 3 is uniformly ^13^C-labeled (denoted *HA*_8_^AN^-^13^*C*_6_-*GlcUA3*) in the absence (*blue*) and presence (*red*) of unlabeled Link_TSG6 protein. *B*, the Link_TSG6/HA_8_^AN^ model showing the proximity of the labeled ring 3 carbons (*magenta*) to Lys^11^ and Tyr^12^ (*orange*); *e.g.* the large downfield shift of the H5 proton (*A*) may be caused by the formation of a salt-bridge between the carboxylate of GlcUA3 with the ϵ-NH_3_^+^ group of Lys^11^.

A HA_8_^AN^ oligomer in which the ring 3 GlcUA was uniformly ^13^C-labeled (denoted HA_8_^AN^-^13^C_6_-GlcUA3) was used to determine the effect of Link_TSG6 binding on the oligosaccharide (*i.e.* close to the position of the kink) and to provide independent information to test the refined model. Fig. 8*A* shows an overlay of ^13^C,^1^H HSQC spectra of the oligomer in the absence (*blue*) or presence (*red*) of unlabeled Link_TSG6, which indicates that the chemical shifts of the ring 3 C-H groups are all perturbed on binding to this protein; it should be noted that because the 8-mer is in more than a 3-fold excess over the protein, peaks are also present at the unbound positions in the latter spectra. Although the C3-H3 resonance only undergoes a relatively small change in chemical shift in the presence of Link_TSG6, the C1-H1, C2-H2, C4-H4, and C5-H5 groups all experience significant perturbations, *i.e.* C1-H1 (^13^C, −1.30 ppm; ^1^H, +0.14 ppm); C2-H2 (+0.07; −0.15); C3-H3 (−0.00; −0.06); C4-H4 (−1.05; −0.01); C5-H5 (−0.31; +0.23).

As can be seen from Fig. 8*B*, the highly perturbed C1-H1 and C5-H5 moieties are on one side of the sugar ring in close proximity to Lys^11^ and Tyr^12^ in the refined Link_TSG6/HA_8_^AN^ model; these amino acids have been found previously to be involved in mediating the interaction with HA based on mutagenesis and NMR analysis (10, 46, 47). Importantly, H5, which experiences the largest perturbation in ^1^H chemical shift on Link_TSG6 binding (+0.23 ppm), is moved downfield, which is consistent with this proton being in proximity to Lys^11^ in the Link_TSG6/HA_8_^AN^ model (see Fig. 2). The C4 carbon is involved in the glycosidic bond linking rings 2 and 3, which is highly kinked such that the Φ/Ψ angles in the family of models, although favorable, occupy a limited region of the energy minima. Thus the distribution of conformations about the 2–3 glycosidic bond are likely to be distinct from those in free solution, which could account for the perturbation of the labeled C4 carbon in the NMR spectra (Fig. 8*A*). Furthermore, the H2 proton experiences a significant up field shift (−0.15 ppm), which perhaps could be explained by the proximity to Phe^70^ (in some models) that has been hypothesized previously to make an aromatic ring stacking interaction with ring 4 (17). Alternatively this perturbation (and the upfield shift changes to H3 and H4) could be due to their proximity to the electron-rich Cys^47^-Cys^68^ disulfide bond.

##### Determining Which HA Oligomers Act as Substrates in Heavy Chain Transfer

**FIGURE 9.**
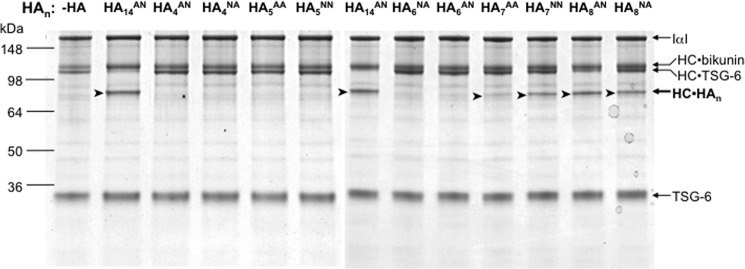
**Determining which HA oligomers act as substrates in heavy chain transfer.** SDS-PAGE analysis was used to determine the relative activities of 10 different HA*_n_* oligomers in the formation of TSG-6-mediated HC·HA*_n_* complexes in comparison with no added HA (−*HA*) and HA_14_^AN^ as a positive control. The relative positions of the HC·HA*_n_* reaction products (*arrowheads*), the bands corresponding to unreacted IαI and free TSG-6, the HC·TSG-6 complexes that act as intermediates in HC transfer, and HC·bikunin byproducts (as defined in Ref. 35) are all indicated on the *right hand side*.

Previously we showed that a HA_14_^AN^ oligosaccharide can act as a substrate in TSG-6-mediated heavy chain transfer, *i.e.* in the formation of a HC·HA complex (35). Here we assessed the relative activities of HA_14_^AN^ compared with the various HA oligomers employed within this study to determine the smallest HA oligosaccharide that can act as a substrate and to see if substrate efficiency correlates with affinity for Link_TSG6. From the representative SDS-PAGE analysis in Fig. 9, it can be observed that the reactions containing HA_8_^AN^, HA_8_^NA^, HA_7_^NN^, or HA_7_^AA^ all gave rise to visible bands of ∼85 kDa, *i.e.* corresponding to HC·HA*_n_*, whereas those with HA 4-, 5-, and 6-mers did not contain any clearly visible bands of this size. Thus, on the basis of these data the minimum size of HA oligosaccharide that can act as a substrate for heavy chain transfer is likely a 7-mer; it should be noted that previous work (on HA*_n_*^AN^ and HA*_n_*^AA^ oligomers) reported in Day *et al.* (71) indicated that HA_5_^AA^ may also act as a weak substrate; however, this finding was not reproduced here. As can be seen from Fig. 9, the HA_8_^AN^ was the preferred substrate for heavy chain transfer, giving the most intense HC·HA*_n_* band (although this was a slightly poorer substrate than the HA_14_^AN^ control), followed by HA_8_^NA^ and HA_7_^NN^ that gave similar amounts of product (*i.e.* approximately half that of HA_8_^AN^). HA_7_^AA^ also acted as a substrate, but transfer of HC onto this oligosaccharide appeared much less efficient (∼10-fold less than for HA_8_^AN^). Interestingly, there appears to be no correlation between substrate efficiency of the 7- and 8-mers and the affinity of these oligomers for Link_TSG6 (see Table 1); *e.g.* the tightest binding oligomer HA_8_^NA^ is a weaker substrate than HA_8_^AN^, and the poor substrate HA_7_^AA^ has a similar affinity to HA_8_^AN^.

## DISCUSSION

Here we have generated a refined model of the Link module from human TSG-6 in complex with a HA octasaccharide based on chemical shift maps for Link_TSG6 in the presence of defined HA oligomers of differing lengths; the use of HA oligosaccharides with GlcNAc at their reducing termini, not available until recently (52), has allowed the identification of novel structural restraints that were used to inform the modeling process. The model was also tested using new sugar reagents, *i.e.* hybrid HA oligosaccharides containing chondroitin disaccharide units and a HA 8-mer where only one sugar ring was isotopically labeled, that have not been described before. The refined model was found to be consistent with ITC and NMR data derived from experiments conducted with 13 distinct oligosaccharides.

Although the conformation and position of HA rings 3–8 are essentially identical to those in our original model (17), the novel interactions observed between ring 1 and the protein have allowed refinement of the Link_TSG6/HA_8_^AN^ complex such that this sugar makes close contacts with both Lys^63^ and His^45^; both of these residues are completely conserved in the TSG-6 sequences from various species characterized to date (*e.g.* see alignment in Blundell *et al.* 60). Mutation of His^45^ has been shown previously to reduce the affinity of the interaction with HA_8_^AN^ by ∼50% (60), which can now be explained by a subtle disruption of the ring stacking interaction between the histidine and GlcNAc2 when it is replaced with serine (Figs. 1 and 2).

Thus, in the new model the association of HA_8_^AN^ with Link_TSG6 is mediated by a combination of salt bridges, ring stacking interactions, hydrogen bonds, and hydrophobic interactions where 7 of the 8 rings make contact with the protein surface (see Fig. 2); to utilize all of these interactions the HA must contact two faces of the Link module, which requires the glycosidic bonds between rings 2 and 3 and between rings 3 and 4, to be highly kinked. In this regard, NMR peaks II and III (*i.e.* arising from Val^62^ and Lys^63^, respectively; Fig. 1) appear to be diagnostic for the presence of this kink in the HA chain (*i.e.* in HA_8_^AN^, HA_8_^NA^, HA_7_^NN^, HA_7_^AA^, and HA_6_^AN^), whereas peak IV may signal the formation of a partial kink, *i.e.* in the oligomers that have a reducing terminal GlcNAc lying in the ring 2 position, *e.g.* HA_6_^NA^, HA_5_^NN^, HA_4_^NA^; peak V likely identifies oligomers that do not kink (HA_5_^AA^, HA_4_^AN^) and thus do not make full contact with the His^45^ and Lys^63^ amino acids (see Fig. 2).

Based on our new modeling, HA_8_^AN^ appears to be the minimum size of oligomer that can make the full complement of interactions with Link_TSG6 (Fig. 2); binding of HA_7_^AA^, although filling the binding site, is entropically unfavorable (Table 1). Moreover, the oligosaccharides with six or fewer sugars all have much lower affinities. The greatly decreased affinity of the hexasaccharides compared with HA_8_^AN^ indicates that multiple interactions along both faces of the Link module surface are needed to generate the energy required to form the pronounced kink in the HA chain. Thus, once the interaction surface is long enough to induce the kink and overcome the “activation energy,” a more favorable binding mode (and thus increased affinity) is attained.

However, it is not currently possible to fully rationalize the affinities of all the various HA oligomers on the basis of their proposed positions within the Link modules binding groove (Fig. 2). For example, determining a mechanism for the unusually high affinity observed for the interaction between Link_TSG6 and HA_8_^NA^ is made more difficult by the observation that this oligosaccharide binds in two registers (see Fig. 2). It is possible that oligomer-specific “end effects” allow better matching of the HA dynamics with the dynamic motion of the protein (*e.g.* in the β4-β5 loop; Ref. 11) leading to a higher affinity; *e.g.* by enhancing the interactions between the reducing terminal GluUA and Arg^81^ (in the lower binding register shown in Fig. 2).

The refined Link_TSG6/HA_8_^AN^ model suggests the formation of three salt bridges, *i.e.* between the carboxylate groups of the oligosaccharide rings 1, 3, and 7 and the side chains of Lys^63^, Lys^11^, and Arg^81^, respectively (Fig. 2). Previous ITC data showed that on average the binding of Link_TSG6 to HA_8_^AN^ only involves ∼1.5 salt bridges (70), suggesting that these ionic interactions are transient. This is consistent with the finding that ionic interactions likely contribute only ∼25% of the free energy of binding at physiological salt strengths (*i.e.* based on ITC measurements over a range of NaCl concentrations; Ref. 70) and also with the conclusion (here and in Refs. 10 and 17) that ring stacking and hydrogen bonds make a major contribution to binding.

**FIGURE 10.**
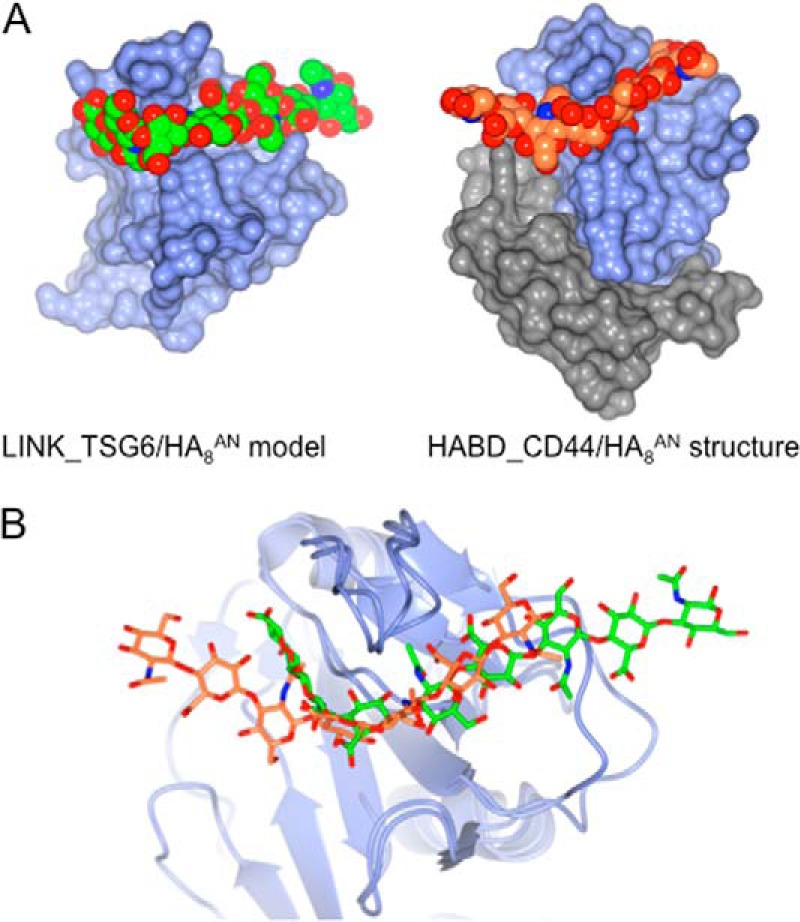
**Similarity of refined Link_TSG6/HA_8_^AN^ model and the CD44/HA_8_^AN^ co-complex.** *A*, a comparison of the Link_TSG6/HA_8_^AN^ model (determined here) with the crystal structure of the HABD from murine CD44 in complex with HA_8_^AN^ (13); the HA is shown in a space-filling representation where carbon atoms are colored *green* or *orange*, respectively. The proteins are represented as solvent-accessible surfaces, where the Link module domains, which are displayed in an equivalent orientation (based on an overlay of their secondary structures), are colored in *light blue*; in CD44, the N- and C-terminal segments that extend the Link module structure to form the larger HABD (12) are colored *gray. B*, an overlay of Link_TSG6/HA_8_^AN^ and HABD_CD44/HA_8_^AN^ with protein ribbons shown in *blue* and the HA shown in *green* (rings 1–8) and orange (rings 2–8), respectively.

As noted already, the HA chain in the refined Link_TSG6/HA_8_^AN^ model is kinked and wraps around the protein surface. From Fig. 10 it can be seen that this is similar to the conformation of HA in the co-crystal structure of HA_8_^AN^ bound to the HABD of CD44 (13). In both of these cases the HA octasaccharide is located in a similarly positioned binding groove on the Link module surface (Fig. 10*A*), although the kink in the CD44/HA complex is somewhat less pronounced (Fig. 10*B*); the HABD of CD44 is composed of additional N- and C-terminal segments that extend the Link module structure (12); however, these do not contribute significantly to the interaction with HA (13). Although there are clearly similarities in the manner in which the CD44 and TSG-6 interact with HA, the molecular details are quite distinct. For example, as described above, in the case of the refined Link_TSG6/HA_8_^AN^ model 7, sugar rings make contact with the protein surface, whereas in CD44 only 5 rings are involved in binding (13). Furthermore, the interaction between Link_TSG6 and HA_8_^AN^ is mediated in part by both ionic and ring stacking interactions (Fig. 2), whereas the binding of HA_8_^AN^ to CD44 does not involve either of these types of interaction but rather is dominated by hydrogen bonds and van der Waals forces (13); the binding site in CD44, however, like TSG-6, does contain a hydrophobic pocket (in a position close to the well defined pocket in Link_TSG6 that is bounded by the Cys^47^-Cys^68^ disulfide) that accommodates a methyl group of the HA. This large difference in the interaction networks of the Link_TSG6/HA_8_^AN^ and HABD_CD44/HA_8_^AN^ complexes probably explains why the binding of HA_8_^AN^ to Link_TSG6 is of much higher affinity than that with HABD_CD44 when determined by ITC under similar conditions (*i.e. K_d_* values of ∼0.2 μm (Table 1) and ∼125 μm (13), respectively). Overall, the TSG-6 Link module and its mode of HA association is expected to be more representative of the Link module family as a whole than CD44 (13, 17).

These differences in the way that TSG-6 and CD44 interact with HA are perhaps not surprising given that the biological roles of these two proteins are so different. CD44 is a cell surface receptor that binds to HA through multiple weak interactions (72, 73), which can for example mediate rolling of leukocytes on the vascular endothelium (74–76); importantly, the low affinity and transient nature of the CD44/HA interaction has been found to be necessary for rolling to occur (77). On the other hand, TSG-6 has been demonstrated recently to form stable complexes with HA that cross-link this polysaccharide leading to the condensation of HA networks (29). This cross-linking may be important for the reorganization of extracellular matrix (*e.g.* at sites of inflammation where TSG-6 is expressed) and is also likely responsible for promoting the association of HA with CD44 on leukocytes (27–29). The recent observation that TSG-6 can decrease nuclear translocation of NF-κB in resident macrophages in a CD44-dependent manner (26) could also be explained by TSG-6-mediated cross-linking of HA (and thus enhanced receptor engagement) rather than by a direct interaction between TSG-6 and CD44 as has been suggested. In the study by Baranova *et al.* (29), both full-length TSG-6 and Link_TSG6 were found to be able to condense and rigidify HA networks even though the isolated Link module domain binds HA more weakly (and non-cooperatively) compared with the intact protein. This condensation of HA could potentially be explained, at least in part, by the pronounced kink that our refined model predicts to be induced in the HA chain on binding to the TSG-6 Link module (*i.e.* leading to an apparent chain shortening).

As described under “Results,” Link_TSG6 can bind to a chondroitin 8-mer with an ∼5-fold lower affinity compared with that of HA_8_^AN^; based on the experiments with the hybrid oligomers, this non-sulfated glycosaminoglycan can be accommodated within the Link module HA-binding groove. The finding that the NMR shift perturbations for Link_TSG6 in the presence of these hybrid oligosaccharides were only subtly different compared with HA_8_^AN^ suggests that TSG-6 is also likely to induce a bent conformation in chondroitin. If this is the case, then the binding of TSG-6 to unsulfated stretches of chondroitin sulfate may be able to condense this glycosaminoglycan (*i.e.* as we have observed for HA; Ref. 29) and could serve to contract the overall domain size of a chondroitin-sulfate proteoglycan. This could have pronounced effects on the organization of extracellular matrices (*e.g.* leading to more condensed matrix structures) and might also enhance the movement of chondroitin-sulfate proteoglycans within tissues, *e.g.* aiding the diffusion of *de novo* synthesized aggrecan out of the chondrocyte pericellular matrix during cartilage remodeling; such activities could be envisaged to contribute to TSG-6 chondroprotective function.

The new model of the Link_TSG6/HA_8_^AN^ complex potentially has implications for understanding the mechanism underlying TSG-6-mediated heavy chain transfer onto HA; TSG-6 has been demonstrated to form covalent complexes with both HC1 and HC2 (*i.e.* HC1·TSG-6 and HC2·TSG-6 (35, 39) that act as intermediates in the formation of HC·HA (35). In this regard a serine residue in the N-terminal region of TSG-6 (*i.e.* Ser^28^ using the numbering for the full-length preprotein (48), in which the first amino acid of the Link_TSG6 would be at sequence position 36) has been shown to form an ester bond with the C-terminal aspartic acid residues of the heavy chains (40); the ester bond in an HC·TSG-6 complex is then transferred from TSG-6 onto the C6 hydroxyl of a GlcNAc sugar in HA (see Ref. 35). Therefore, it is conceivable that HA recognition during the HC transfer reaction could involve the binding of HA to the Link module of TSG-6 (*i.e.* where the HA adopts the conformation in our model). However, this scenario seems unlikely given that there was no correlation between the substrate activities of various HA oligomers and their affinities for Link_TSG6 (*e.g.* HA_7_^AA^ was a much poorer substrate than HA_8_^AN^ while having similar binding constants; Table 1 and Fig. 9). This observation suggests that the binding site for HA in the context of an HC·TSG-6 complex may be distinct from that in free TSG-6. Consistent with this hypothesis, we have found that the interaction between TSG-6 and IαI (and the formation of the HC·TSG-6 complex) inhibits the binding of HA to TSG-6 (28); this prevents TSG-6-mediated cross-linking of HA and can abolish the enhancement of HA binding to cell surface CD44. Thus the interaction of HC with TSG-6 likely occludes the HA-binding site in TSG-6 or stabilizes the Link module in its closed conformation; either way this would prevent some or all of the HA-binding residues described here from interacting with HA. In this regard we have found that a mutant of full-length TSG-6 (where Tyr^94^ (equivalent to Tyr^59^ in Link_TSG6) was mutated to phenylalanine), with greatly impaired HA-binding activity,^6^ is able to mediate HC transfer (78). Further work is now necessary to determine whether the HA-binding groove that we have defined in the Link_TSG6/HA_8_^AN^ complex plays any role in the formation of HC·HA complexes.

In summary, we have used experimental data derived from a series of 13 defined and distinct oligosaccharides to build a refined model of an HA octasaccharide in complex with the TSG-6 Link module domain. This model has provided new insights into the molecular basis of HA/protein interactions and is helpful in furthering our understanding of the functional role of TSG-6 in extracellular matrix reorganization.

## Footnotes

6 M. R. Rugg and A. J. Day, unpublished data.

## Footnotes

5 The abbreviations used are:

HA: hyaluronan
GlcUA: d-glucuronic acid
ECM: extracellular matrix
HABD: hyaluronan-binding domain
IαI: inter-α-inhibitor
HC: heavy chain from IαI
HSQC: heteronuclear single quantum coherence
ITC: isothermal titration calorimetry
Link_TSG6: recombinant Link module from human TSG-6
Tricine: *N*-[2-hydroxy-1,1-bis(hydroxymethyl)ethyl]glycine.
